# Basic and clinical immunology – 3036. Investigation of peripheral follicular helper T-cells in primary Sjögren's syndrome

**DOI:** 10.1186/1939-4551-6-S1-P210

**Published:** 2013-04-23

**Authors:** Krisztina Szabo, Gabor Papp, Sandor Barath, Edit Gyimesi, Antonia Szanto, Margit Zeher

**Affiliations:** 1 Dept. of Medicine, University of Debrecen, MHSC, Hungary; 2 Biotest Hungaria Kft., Hungary

## Background

The exact mechanisms behind the immune disturbances in primary Sjögren´s syndrome (pSS) are still not known in details, but evidence suggests that abnormal T- and B-cell activation plays a critical role in the disease development. Recent studies emphasized the important role of follicular helper T (T_FH_) cells, which contribute to B-cell proliferation and differentiation, as well as antibody production. The aim of this study was to investigate the possible role of T_FH_ cells in the pathogenesis of pSS, by analyzing a wide spectrum of immune-competent cells and serological markers with a special emphasis on clinical symptoms of the disease.

## Methods

We enrolled 50 patients suffering from pSS [25 with extraglandular manifestations (EGMs) and 25 without EGMs] and 16 healthy individuals as controls in the study. Peripheral lymphocyte subpopulations were determined by flow cytometry, circulating cytokines and autoantibodies were quantified by ELISA technique. Statistical analysis was performed using GraphPad Prism 5 software and SPSS version 16.0.

## Results

Patients with pSS showed elevated ratio of peripheral T_FH_ cells. However, when we divided patients into two groups defined by the presence or absence of EGMs, only patients with EGMs had significant differences, while values of patients without EGMs were similar to healthy controls. We found a significant positive correlation between activated T and T_FH_ cell percentages as well as between the Tr1 and T_FH_ cells. Significant negative correlations were observed between IgM memory B and T_FH_ cell values and between IgG memory B and T_FH_ cell proportions. Elevated T_FH_ percentages were observed in the anti-SSA positive patients, compared to autoantibody negative patients and healthy controls. Moreover, the percentages of T_FH_ cells were significantly elevated in patients with higher IL-12 and IL-21 levels.

## Conclusions

Our data suggest that percentages of peripheral T_FH_ cells are increased in pSS, especially in patients suffering from a systemic, more pronounced course of disease (pSS with EGMs). Moreover, the elevated T_FH_ cell percentages correlates with activation of immune system, proportions of memory B cells and titers of autoantibodies, which implies that T_FH_ cells may play an important role in the disease development.

